# Understanding the role of overdose witnesses: responses and characteristics of people who inject drugs in Oakland and San Francisco

**DOI:** 10.1186/s12954-026-01436-8

**Published:** 2026-03-08

**Authors:** Bow Suprasert, Raul Ruiz, Jose Francisco, Paavani Lella, Iris R. O’Neal, Gueslyn Velasquez, Glenda N. Baguso, Glenn-Milo Santos, Erin C. Wilson, Eileen F. Dunne, Willi McFarland

**Affiliations:** 1 San Francisco Department of Public Health, Center for Public Health Research, San Francisco, CA 94102-6033 USA; 2 San Francisco Department of Public Health, Center on Substance Use and Health, San Francisco, CA USA; 3 Department of Epidemiology and Biostatistics, University of California, San Francisco, CA USA; 4 Alameda County Public Health Department, San Leandro, CA USA

**Keywords:** Overdose witness, Opioid overdose, People who inject drugs, PWID

## Abstract

**Background:**

Overdose is a significant cause of mortality in the United States. People who inject drugs (PWID) are uniquely positioned to witness and reverse overdoses. This study assesses overdose responses and factors associated with witnessing overdose among PWID.

**Methods:**

Data originate from baseline surveys for a longitudinal study of PWID in Oakland and San Francisco, California, collected between April 2024 and March 2025. Multivariable logistic regression identified factors associated with witnessing overdoses.

**Results:**

Of 597 PWID, 464 (77.7%) witnessed at least one overdose in the past 3 months, with an average of 13.4 (SD = 28.2) and a median of 6 (IQR = 3–15) overdoses. Among those, 30 (6.5%) reported only calling 911, 50 (10.8%) reported taking no action, 172 (37.2%) reported only using naloxone to reverse overdoses, and 210 (45.5%) used naloxone in conjunction with calling 911. The adjusted odds of witnessing overdoses were 2.2 times higher among unsheltered PWID (vs. sheltered, *p* < 0.001), 3.1 times higher among those in San Francisco (vs. Oakland, *p* < 0.001), 4.6 times higher among those who experienced non-fatal overdose themselves (vs. not, *p* = 0.002), 2.8 times higher among those sharing needles (vs. not, *p* = 0.015), and 1.9 times higher among those injecting fentanyl (vs. did not, *p* = 0.006) in the past 3 months.

**Conclusions:**

Nearly four in five PWID in Oakland and San Francisco recently witnessed an overdose. Witnesses are at greater risk of non-fatal overdose and may be the first to respond when those they inject with overdose. Targeting naloxone distributions to PWID who are characteristically more likely to witness overdoses may prevent additional drug-related deaths.

## Background

Drug overdose is a significant cause of mortality, with over 80,000 deaths in the United States in 2024 [1]. Opioids, including synthetic opioids such as fentanyl, were involved in more than half of these drug-related deaths [1]. That same year, San Francisco, California, recorded 635 overdose deaths, with fentanyl involved in over 70% of these deaths [2]. About 23% of these overdose deaths occurred in public places, and 42% occurred in low-income neighborhoods [2]. In May 2025, Santa Clara County, located less than 100 miles from San Francisco, reported its first death from a counterfeit pill that contained carfentanil, a new synthetic opioid that is 100 times more potent than fentanyl [3].

Currently, two key preventive measures, fentanyl test strips and naloxone, are available to help save lives. Fentanyl test strips detect fentanyl contamination in drugs, particularly in counterfeit drugs that can be mistaken for depressants, such as Xanax, or stimulants, such as cocaine or methamphetamine [3, 4]. Naloxone is a life-saving medication that can be sprayed into a person’s nasal cavity to reverse the effects of an opioid overdose and prevent death. One standard naloxone kit comes with two doses, and it is recommended to administer each dose every 2 to 3 min until the person can breathe on their own [3, 5]. Naloxone is safe to use even if the unconscious person is not experiencing an opioid overdose [5]. Multiple public health agencies and harm reduction organizations throughout the San Francisco Bay Area offer free fentanyl test strips and naloxone to individuals at risk of overdose and any active bystanders [6, 7].

About two-thirds of overdose deaths in the United States occurred in the presence of bystanders, although they may not always know how to respond [8]. A recent simulation study found that expanding naloxone distribution, combined with a 60% increase in the proportion of overdoses witnessed, could reduce overdose deaths by up to 37.4% [6]. People who use drugs, particularly males, unhoused, and have overdosed, were more likely to witness overdoses [9]. However, studies have shown that witnesses may hesitate to call 911 due to fear of arrest for drug possession, drug paraphernalia, drug use, or other legal concerns [10–12].

Limited data on witnessing overdoses are available from community-collected samples of people who inject drugs (PWID) in San Francisco and neighboring Oakland. Their presence on the street or during injection with others puts them in a unique position to witness, intervene, and potentially save the lives, including those they inject with [9]. Understanding which individuals within PWID communities witness overdoses and how they respond is key to optimizing naloxone and directing other overdose prevention resources where they can most effectively reduce drug-related overdoses [13].

## Methods

### Sampling design and recruitment

The Brief Longitudinal Incidence Sentinel Surveillance (BLISS) study is a cohort of 600 PWID in Oakland (*N* = 300) and San Francisco (*N* = 300), California, United States. BLISS was a collaborative effort between the Alameda County Public Health Department and the San Francisco Department of Public Health to capture HIV-related events, including patterns of substance use and experiences of overdose. Eligibility criteria were (1) aged 18 years or older; (2) injected in the past three months; (3) reside in Oakland or San Francisco, California; and (4) speak English or Spanish. PWID were recruited via Starfish sampling. Details on Starfish Sampling are discussed in length elsewhere [14]. In brief, Starfish Sampling combines the peer-referral approach of respondent-driven sampling (RDS) with venue-based outreach recruitment, as in time-location sampling (TLS). In the BLISS Study, TLS was conducted at five venues: encampment areas, public parks, public libraries, community health centers, and harm reduction sites in Oakland and San Francisco, California. The RDS component entailed peer referral by other participants, regardless of recruitment location.

Participants provided written informed consent before an interviewer-administered baseline survey and a rapid HIV test. Participants were provided with a $50 prepaid debit card for completing the baseline survey and HIV test. Participants received $10 for each successful recruit, with no limit on the number of recruits. A total of 8 follow-up surveys are conducted quarterly and distributed electronically via URL links sent via SMS and/or email. Participants received $20 for completing each follow-up survey. The present study reports only the cross-sectional baseline data collected between April 2024 and March 2025.

### Measures

Basic demographic characteristics (e.g., age, race/ethnicity, education, employment status, monthly income, living situation) were collected at baseline. Some variables were categorized to improve interpretability. For example, age was categorized into age groups (i.e., 18–29, 30–39, 40–49, 50–59, and 60 + years). Monthly income was collected using the following categories: <$1,000 USD, $1,000-$1,999 USD, $2,000-$2,999 USD, $3,000-$3,999 USD, $4,000-$4,999, and ≥$5,000. Incomes of more than $2,000 USD were grouped together. For context, a median household income (in 2023 dollars) is $97,369 USD, which translates to a monthly income of $8,114 USD in Oakland, California, and $141,446 USD or $11,787 USD per month in San Francisco, California [15]. While not a precise measure, a monthly income of less than $2,000 USD is generally considered extremely low in high-cost areas such as Oakland and San Francisco, California. Additionally, a monthly income below $1,000 USD falls well below the 2024 federal poverty level threshold of $1,255 USD [16].

The timeframe for all other variables was in the past 3 months. Homelessness and incarceration were asked as, “In the past 3 months, how many nights did you sleep on the street?” and “In the past 3 months, how many times have you been incarcerated?”. Responses were dichotomized into yes/no for analytic simplicity and interpretability. Injection behaviors (e.g., number of years injected, daily injection, etc.) and access to harm reduction services were also collected. Needle sharing in the past 3 months was determined based on two questions: (1) “In the past 3 months, did you use a needle after someone else injected with it?” and (2) “In the past 3 months, did you give your needle to someone else after you had already injected with it?”. A yes response to either question was classified as having shared needles. Each drug (i.e., heroin, methamphetamine, non-prescribed painkiller, fentanyl, crack cocaine, powder cocaine, xylazine or tranq, speedball - a combination of heroin and cocaine, goofball - a combination of heroin and methamphetamine, new speedball - a combination of fentanyl and methamphetamine, and other with an option to specify) was assessed separately. We report four drugs most commonly injected among our PWID sample: heroin, methamphetamine, fentanyl, and new speedball.

Experiences of overdose and overamp were assessed independently and jointly. In other words, we created a composite categorical variable that classified participants into four mutually exclusive groups: those who experienced neither overdose nor overamp, those who experienced overdose only, those who experienced overamp only, and those who experienced both overdose and overamp in the past 3 months. An overdose was defined as passing out, turning blue, or stopping breathing, often from overusing opioids. Overamp was defined as having a fast and racing heart, tightening in the chest, limb jerking, nausea or vomiting, extreme sweating or high temperature, convulsions, seizures, cardiac arrest, or stroke, often from overusing stimulants.

### Outcome measure

Witnessing overdoses was asked as “In the past 3 months, how many overdose(s) have you witnessed?”. Responses were dichotomized into yes/no for further analysis. Those refusing to respond were excluded from the analysis, resulting in a final analytical sample of *N* = 597 (*N* = 298 in Oakland and *N* = 299 in San Francisco). Three follow-up questions were asked to assess overdose responses: 1) “Out of the number of overdose(s) that you witnessed, how many times have you reversed an overdose using naloxone/Narcan?” and 2.) “In the past 3 months, how many times have you called 911 when someone was overdosing?”. If participants responded with 0 (zero) to the 911 call question, “What are the reasons that you did not call 911 when someone was overdosing?” was asked. Answer choices were (1) I did not know how to ask for help; (2) I did not have a phone or phone service to make a call; (3) Someone else already called; (4) I was reversing the overdose; (5) I asked someone else to call; (6) I did not think law enforcement could help; (7) I was afraid of being arrested; (8) I did not think a 911 call was needed; (9) Other, with an option to specify. Multiple responses were allowed.

### Analysis

We report descriptive statistics as counts and proportions for categorical variables and means (SD)/medians (IQR) for continuous variables. Chi-squared, Fisher’s Exact, T-test, Wilcoxon Rank-Sum, and Pearson Correlation tests were conducted to identify factors associated with witnessing overdoses among PWID in Oakland and San Francisco. Following these preliminary analyses, bivariate logistic regressions were conducted to quantify the magnitude and direction of the associations identified by the bivariate analyses. Multivariable logistic regression was then developed to evaluate the independent effect of each variable. Variables with significant associations (P-value < 0.05) in the bivariate models were considered as candidates. Variables with sparse data, specifically those with at least one cell containing fewer than 5 observations, were excluded to avoid issues with perfect prediction. This criterion led to the exclusion of age groups, composited overdose/overamp, and harm reduction supplies receival variables in the multivariable model. These data are presented in their original categories rather than recategorized to maintain transparency and interpretability.

Collinearity diagnostics were also performed to prevent overfitting and to address multicollinearity among the remaining candidate variables. Variables with small eigenvalues (λ close to 0) or large condition numbers (κ > 10) were removed to help stabilize coefficient estimates. Methamphetamine injection was identified as one such variable and thus excluded from the final model. Overdose was prioritized over overamp in the multivariable analysis as it provides greater interpretability and public health relevance. Considering an association between the two measures, we also examined non-fatal overamp as the primary covariate in place of overdose; results were similar in magnitude and direction. Backward stepwise regression technique was then used to identify a model with the lowest Akaike Information Criterion (AIC). The final model (AIC = 549.82, df = 7) was reported. The final model adjusted for key covariates, including birth sex, homelessness status, city of residence, self-reported history of non-fatal overdose, needle sharing behaviors, and fentanyl injection. Significant value was set at α = 0.05. All analyses were conducted using STATA 18 [17].

### Ethical considerations

The study was reviewed and approved by the Institutional Review Board (IRB) of the University of California, San Francisco (IRB#23-40130). All participants provided written informed consent. Data were reported anonymously.

## Results

Of 597 PWID, 464 (77.7%) witnessed at least one overdose in the past 3 months (Table 1). By city, 199 (42.9%) of PWID witnessing overdoses reside in Oakland, while 265 (57.1%) reside in San Francisco, California. The number of overdoses witnessed ranged between 1 and 450, with an average of 13.4 (SD = 28.2) and a median of 6 (IQR = 3–15) overdoses witnessed in the past 3 months. The number of overdoses witnessed differed significantly between Oakland and San Francisco, California. In Oakland, PWID reported witnessing an average of 8.4 (SD = 13.0) and a median of 4 (IQR = 2–8) overdoses, while in San Francisco, they reported an average of 17.1 (SD = 35.2) and a median of 9 (IQR = 4–20) (t-value (462) = -3.32, *p* = 0.001; z-value = -5.44, *p* < 0.001).

**Table 1:** Demographic characteristics of people who inject drugs witnessing an overdose in the past 3 months in Oakland and San Francisco, BLISS study, *N* = 597

Characteristics	Total *N* = 597 (col%)	Did not witness an overdose *N* = 133 (row%; 22.3)	Witnessed an overdose *N* = 464 (row%; 77.7)	X^2^ value	*P*-value
Age group in years					
18–29	17 (2.9)	0 (0)	17 (100)	10.18	0.037
30–39	163 (27.3)	34 (20.9)	129 (79.1)		
40–49	194 (32.5)	38 (19.6)	156 (80.4)		
50–59	146 (24.5)	37 (25.3)	109 (74.7)		
60+	77 (12.9)	24 (31.2)	53 (68.8)		
Sex at birth					
Male	406 (68.0)	80 (19.7)	326 (80.3)	4.85	0.028
Female	191 (32.0)	53 (27.7)	138 (72.3)		
Race/ethnicity				8.59	0.072
White	228 (38.3)	41 (18.0)	187 (82.0)		
Black/African American	229 (38.5)	64 (28.0)	165 (72.0)		
Hispanic	32 (5.4)	5 (15.6)	27 (84.4)		
Multiracial	21 (3.5)	6 (28.6)	15 (71.4)		
Other	85 (14.3)	16 (18.8)	69 (81.2)		
Born in the United States				3.07	0.089
No	25 (4.2)	2 (8.0)	23 (92.0)		
Yes	572 (95.8)	131 (22.9)	441 (77.1)		
Education				5.23	0.156
Less than high school	115 (19.3)	32 (27.8)	83 (72.2)		
High school graduate or	242 (40.6)	57 (23.5)	185 (76.5)		
GED					
Some college	185 (31.0)	35 (18.9)	150 (81.1)		
College graduate or higher	54 (9.1)	8 (14.8)	46 (85.2)		
Employment				6.37	0.095
Unemployed	344 (57.7)	65 (18.9)	279 (81.1)		
Unable to work due to health	161 (27.0)	46 (28.6)	115 (71.4)		
Employed	54 (9.1)	14 (25.9)	40 (74.1)		
Other (e.g., students, retired)	37 (6.2)	8 (21.6)	29 (78.4)		
Monthly income				3.74	0.154
Less than $1000 USD	374 (62.6)	75 (20.0)	299 (80.0)		
$1000 - $1999 USD	155 (26.0)	43 (27.7)	112 (72.3)		
$2000 USD or more	68 (11.4)	15 (22.1)	53 (77.9)		
Had a cellphone				1.31	0.253
No	134 (22.5)	25 (18.7)	109 (81.3)		
Yes	463 (77.5)	108 (23.3)	355 (76.7)		
Homelessness, past 3 months				20.11	< 0.001
No	205 (34.4)	67 (32.7)	138 (67.3)		
Yes	391 (65.6)	65 (16.6)	326 (83.4)		
Had belongings swept up, past 3 months				30.63	< 0.001
No	322 (54.1)	100 (31.1)	222 (68.9)		
Yes	273 (45.9)	33 (12.1)	240 (87.9)		
Incarcerated, past 3 months				3.87	0.049
No	539 (90.3)	126 (23.4)	413 (76.6)		
Yes	58 (9.7)	7 (12.1)	51 (87.9)		
Current residency				41.15	< 0.001
Oakland	298 (49.9)	99 (33.2)	199 (66.8)		
San Francisco	299 (50.1)	34 (11.4)	265 (88.6)		
Overdosed, past 3 months				15.05	< 0.001
No	513 (85.9)	128 (25.0)	385 (75.0)		
Yes	84 (14.1)	5 (6.0)	79 (94.0)		
Overamped, past 3 months				9.82	0.002
No	466 (78.1)	117 (25.1)	349 (74.9)		
Yes	131 (21.9)	16 (12.2)	115 (87.8)		
Overdose/Overamp, past 3 months				22.38	< 0.001
Neither overdosed nor overamped	417 (69.8)	114 (27.3)	303 (72.7)		
Overdosed only	49 (8.2)	3 (6.1)	46 (93.9)		
Overamped only	96 (16.1)	14 (14.6)	82 (85.4)		
Both overdosed and overamped	35 (5.9)	2 (5.7)	33 (94.2)		
Injection behavior					
Number of years injected					
Average (SD)	12.8 (10.4)	12.5 (11.2)	12.9 (10.2)	- 0.36	0.722
Median (IQR)	10 (4–20)	10 (3–20)	10 (5–20)	- 1.04	0.299
Injected every day, past 3 months				1.89	0.170
No	274 (45.9)	68 (24.8)	206 (75.2)		
Yes	323 (54.1)	65 (20.1)	258 (79.9)		
Shared needle, past 3 months				15.51	< 0.001
No	499 (83.6)	126 (25.3)	373 (74.7)		
Yes	98 (16.4)	7 (7.1)	91 (92.9)		
Injected heroin, past 3 months				0.06	0.803
No	284 (47.6)	62 (21.8)	222 (78.2)		
Yes	313 (52.4)	71 (22.7)	242 (77.3)		
Injected methamphetamine, past 3 months				9.89	0.002
No	185 (31.0)	56 (30.3)	129 (69.7)		
Yes	412 (69.0)	77 (18.7)	335 (81.3)		
Injected fentanyl, past 3 months				23.74	< 0.001
No	334 (56.0)	99 (29.6)	235 (70.4)		
Yes	263 (44.0)	34 (12.9)	229 (87.1)		
Injected a combination of methamphetamine and fentanyl, past 3 months				4.28	0.039
No	494 (82.7)	118 (23.9)	376 (76.1)		
Yes	103 (17.3)	15 (14.6)	88 (85.4)		
Access to harm reduction services					
Received harm reduction supplies, past 3 months				10.40	0.001
No	9 (1.5)	6 (66.7)	3 (33.3)		
Yes	588 (98.5)	127 (21.6)	461 (78.4)		
Used fentanyl test strips, past 3 months				3.13	0.077
No	381 (63.9)	93 (24.4)	288 (75.6)		
Yes	215 (36.1)	39 (18.1)	176 (81.9)		

Witnesses were more likely to be younger (18–29 years old 100.0% vs. among ≥ 30 years old 77.1%, X^2^ = 5.02, *p* = 0.025), male sex at birth (80.3% vs. female 72.3%, X^2^ = 4.85, *p* = 0.028), experienced homelessness (83.4% vs. did not 67.3%, X^2^ = 20.11, *p* < 0.001), had their belongings swept up by the city (87.9% vs. did not 68.9%, X^2^ = 30.63, *p* < 0.001), and were incarcerated (87.9% vs. were not 76.6%, X^2^ = 3.87, *p* = 0.049) in the past 3 months. Witnesses were also more likely to reside in San Francisco (88.6% vs. Oakland 66.8%, X^2^ = 41.15, *p* < 0.001), experienced non-fatal overdose (94.0% vs. 75.0%, X^2^ = 15.05, *p* < 0.001), and experienced non-fatal overamp (87.8% vs. 74.9%, X^2^ = 9.82, *p* = 0.002). There was no correlation between the number of times PWID witnessed overdoses and the number of times PWID experienced non-fatal overdoses (*p* = 0.716) or non-fatal overamp (*p* = 0.676). Of note, 42.4% of PWID reporting non-fatal overdose experienced non-fatal overamp, whereas 27.3% of PWID reporting non-fatal overamp experienced non-fatal overdose (*p* < 0.001).

In regard to injection behavior, there were no differences in number of years injecting (Mean = 12.9, SD = 10.2 vs. Mean = 12.5, SD = 11.2, t = -0.36, *p* = 0.722) or in daily drug injection (79.9% vs. 75.2%, X^2^ = 1.89, *p* = 0.170) in the past 3 months between those witnessing overdoses and those who did not. However, PWID who shared needles with others (92.9% vs. 74.7%, X^2^ = 15.51, *p* < 0.001), injected methamphetamine (81.3% vs. 69.7%, X^2^ = 9.89, *p* = 0.002), injected fentanyl (87.1% vs. 70.4%, X^2^ = 23.74, *p* < 0.001), and injected a combination of methamphetamine and fentanyl (85.4% vs. 76.1%, X^2^ = 4.28, *p* = 0.039) were more likely to witness overdoses than those who did not. Nearly all witnesses (99.4%) received harm reduction supplies such as naloxone, while fewer than half (37.9%) reported using fentanyl test strips in the past 3 months.

Of 464 PWID who witnessed overdoses, 382 (82.7%) used naloxone to reverse an overdose. Among them, the average number of times naloxone was used to reverse an overdose in the past 3 months was 10.2 times (SD = 25.1), with a median of 4 (IQR = 2–10). Meanwhile, 241 (52.2%) PWID reported calling 911 when witnessing overdoses, with an average of 5.2 times (SD = 9.1) and a median of 3 times (IQR = 1–5) that calls were made in the past 3 months. The proportion of witnesses who used naloxone or called 911 did not vary by study location (79.2% in Oakland vs. 85.3% in San Francisco, X^2^ = 2.93, *p* = 0.087; 49.5% in Oakland vs. 54.2% in San Francisco, X^2^ = 0.99, *p* = 0.320, respectively).

**Fig. 1:**
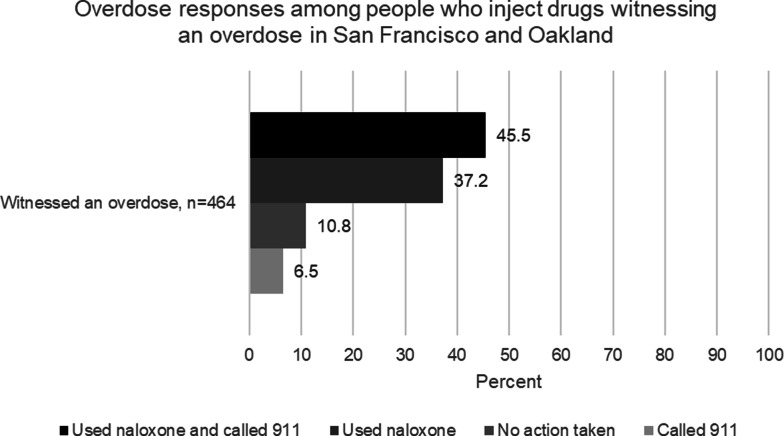
Overdose responses among people who inject drugs, witnessing an overdose in the past 3 months in Oakland and San Francisco, BLISS study

When assessing reasons for not calling 911 when witnessing overdoses, nearly half (46.4%) reported that someone else had already called 911, while about one-third (33.8%) did not think that a 911 call was needed. Other reasons included being the one reversing the overdose (21.6%), not having a phone or phone service to make a call (10.4%), believing that a 911 call would not help (5.0%), fear of arrest (3.6%), having asked someone else to call (2.3%), and other reasons (0.9%). Of 464 PWID witnessing overdoses, 30 (6.5%) reported only calling 911, 50 (10.8%) reported taking no action, 172 (37.2%) reported only using naloxone to reverse overdoses, and 210 (45.5%) used naloxone in conjunction with calling 911 (Fig. 1).

Results of bivariate and multivariable logistic regressions are shown in Table 2. After adjusting for all candidate variables, the adjusted odds of witnessing overdoses were 2.17 times higher among PWID who experienced homelessness in the past 3 months (vs. did not, *p* < 0.001, 95% CI: 1.41–3.33), and 3.12 times higher among PWID residing in San Francisco (vs. Oakland, *p* < 0.001, 95% CI: 1.97–4.92). Additionally, the adjusted odds were 4.56 times higher among PWID who experienced non-fatal overdose (vs. did not, *p* = 0.002, 95% CI: 1.76–11.81), 2.80 times higher among PWID who shared needles (vs. did not, *p* = 0.015, 95% CI: 1.22–6.43), and 1.91 times higher among PWID who injected fentanyl (vs. did not, *p* = 0.006, 95% CI: 1.20–3.04) in the past 3 months.

**Table 2:** Results of logistic regression on witnessing overdoses among people who inject drugs in the past 3 months in Oakland and San Francisco, BLISS study, *N* = 597

	OR	95% CI	aOR	95% CI
Sex at birth				
Male	1.57	1.05–2.34	1.37	0.88–2.12
Female	Ref	–	Ref	–
Homelessness, past 3 months				
No	Ref	–	Ref	–
Yes	2.44	1.64–3.61	2.17	1.41–3.33
Current residency				
Oakland	Ref	–	Ref	–
San Francisco	3.88	2.52–5.97	3.12	1.97–4.92
Overdosed, past 3 months				
No	Ref	–	Ref	–
Yes	5.25	2.08–13.26	4.56	1.76–11.81
Shared needle, past 3 months				
No	Ref	–	Ref	–
Yes	4.39	1.98–9.72	2.80	1.22–6.43
Injected fentanyl, past 3 months				
No	Ref	–	Ref	–
Yes	2.84	1.85–4.36	1.91	1.20–3.04

OR: odds ratio; aOR: adjusted odds ratio; adjusted for all variables shown on table

## Discussion

Nearly four in five PWID in Oakland and San Francisco, California, have witnessed overdoses. On average, PWID in our sample witnessed 13 overdoses in the past 3 months; that is, 4.3 overdoses per month. PWID in San Francisco reported witnessing an average of 17 overdoses in the past 3 months; that is, 5.7 overdoses per month, while PWID in Oakland reported witnessing an average of 8 overdoses in the past 3 months, or about 2.7 overdoses per month. Differences in witnessed overdoses may reflect how dispersed PWID networks are relative to city size. San Francisco’s population density (827,526 people in 46.7 square miles) is nearly double that of Oakland (443,554 people in 55.8 square miles), which could affect how PWID are aggregated and how overdoses are witnessed [15]. Future research should examine structure, dynamics, and contextual differences in PWID networks between two cities to inform more effective harm reduction and overdose prevention strategies.

While there is no direct explanation for the differences observed, the number of 911 calls following an overdose may offer some insights. In Alameda County, where Oakland is located, an average of 4 overdose-related emergency calls were made per day between 2019 and 2021 [18]. In San Francisco, overdose-related emergency calls averaged 12 per day (4,557 total) in 2023 and 9 per day (3,276 total) in 2024 [19]. The lack of updated data in Alameda County limits our ability to make direct comparisons, particularly given that 2019–2021 coincided with the height of the COVID-19 pandemic. Pandemic-related disruptions to healthcare access, emergency response, and drug-use patterns may have influenced overdose incidence, 911 calls, and emergency service utilization. Nevertheless, in San Francisco, where city-level data correspond with our data collection period, the reported number of 911 calls likely underestimates the true burden of overdoses among PWID. Many overdoses might be managed privately or with peer-administered naloxone, as PWID and other communities at high risk of overdose may not always call 911 for help [20].

There are several reasons why PWID may hesitate to call 911. Our results indicate that the primary reason is that someone else has already called, suggesting that participants are likely visible to the public or have strong social ties within their PWID communities. A lack of a phone may also affect PWID’s ability to call 911. While most of our PWID sample had cellphones at baseline, many had their phones stolen, lost, or out of service by the time of their quarterly follow-up. Many people who use drugs have also had poor experiences with EMS personnel and police officers when seeking help, including being labeled with derogatory terms, searched before receiving naloxone, or arrested despite protections under Good Samaritan laws [11, 12, 21]. These experiences contribute to a deep-rooted lack of trust in law enforcement, which further discourages PWID from calling when their peers overdose [11, 18].

Following a Supreme Court ruling to criminalize unhoused individuals in June 2024, city officials have been citing, arresting (and releasing), and sweeping encampments in Oakland and San Francisco, California [22–24]. Most PWID in our study experienced homelessness. These street sweeps may not only exacerbate street-based trauma but also further erode trust in law enforcement among PWID communities [25]. In turn, compounded trauma and diminished trust could contribute to rising in overdose deaths, as observed in 2025 [4, 26]. Providing trauma-informed training to first responders and restricting law enforcement presence at street sweeps and overdose emergencies could increase the likelihood that PWID communities will call 911 for help [18, 25].

In this context, our data can offer insights into the response to the current opioid epidemic. First, we found that nearly half of PWID used naloxone in conjunction with calling 911 when witnessing an overdose. This finding is consistent with a prior study in New York City, which similarly suggested that increased availability of naloxone may not necessarily decrease 911 calls [10]. For this reason, PWID who inject drugs together are uniquely positioned to be both overdose victims and first responders [14]. This dual role PWID has may help reduce the number of fatal overdoses, although it may not necessarily decrease incidents of non-fatal overdoses. A qualitative study is needed to understand whether PWID tend to inject similar drugs and dosages when injecting together, as different drugs and dosages can have varying effects on cognition and their ability to promptly respond to an overdose [24].

Given that many PWID participants reported injecting non-opioids and experiencing non-fatal overamp, either alone or alongside non-fatal overdose, interventions targeting opioid-related overdoses alone may be increasingly insufficient. With evolving stimulant use patterns, future prevention and harm reduction strategies may consider broader, non-opioid-specific approaches, as non-opioids can reduce the effectiveness of naloxone and further complicate overdose responses [27]. Such strategies could address upstream social determinants of health, such as housing instability and post-incarceration reintegration, that contribute to increased PWID’s vulnerability to both experiencing and witnessing overdoses [28]. Safe consumption sites may also provide professional supervision and timely responses to complicated drug-related events [29].

In the meantime, the ongoing distribution of naloxone remains essential, particularly on the streets and in low-income neighborhoods where most PWID reside. Many efforts by the state and local governments have been mobilized to address our overdose epidemic. Specifically, the San Francisco Department of Public Health has nearly quintupled naloxone distribution to community partners, from 41,972 doses in 2021 to 202,145 doses in 2024 [30]. The DOPE Project, Project FRIEND, and NDP have been instrumental in expanding naloxone distribution to shelters, healthcare providers, paramedics, and other key stakeholders [6, 7]. Sustained and expanded funding will be critical to ensure these efforts continue to reduce devastating overdose deaths in our cities.

At the same time, PWID may be generally well-prepared to respond to overdoses, as repeated experiences of witnessing and reversing them have normalized these distressing events [11, 18]. Although our study did not directly assess knowledge of overdose signs, past research and current anecdotal evidence suggest that PWID were aware of the signs of overdose and knew how to respond [24]. PWID may also be highly willing to intervene in an overdose even if they do not know the person well [31]. However, willingness to intervene in an overdose does not necessarily mean the response is appropriate or effective. Previous studies have found that instead of calling 911, PWID may attempt to respond to an overdose by injecting salt, slapping, shaking, or splashing cold water on the individual [31]. Targeted education on overdose response and risk remains critical, given the increased overdose risk associated with drug injection [32].

Reaching and engaging the most affected PWID communities can be vital to effectively tackling the overdose crisis in our cities. Our results indicate that certain groups within the PWID community may be particularly important to prioritize for naloxone distribution. These include young PWID, experiencing homelessness, living in San Francisco, experiencing non-fatal overdose, experiencing non-fatal overamp, sharing needles, or injecting fentanyl. Our findings are similar to previous research from Baltimore and New York City, which found that witnesses were more likely to use heroin [9, 33]. Our results likely reflect the local drug market, which has been flooded with fentanyl, and more recently, a new synthetic opioid called carfentanil [3]. Targeting naloxone distribution efforts toward sub-groups within the PWID population could decrease overdose-related deaths in our cities.

### Limitations

Several limitations should be acknowledged. First, the data presented are from a baseline assessment and are cross-sectional in nature. Therefore, causal inferences cannot be drawn. Second, our reported measures are based on the past 3 months, which may exclude events that occurred further in the past. Third, our question about witnessing overdoses did not differentiate between fatal and non-fatal events, nor did it specify whether the overdose occurred in a public or private setting. However, since most participants reported taking action (e.g., administering naloxone or calling 911), we assume that most witnessed overdoses did not result in fatalities. Nevertheless, witnessing an overdose, whether fatal or non-fatal, can be profoundly traumatic, particularly when such events are recurrent and occur within close social networks [34, 35]. With over half of the PWID sample experiencing homelessness, many of the overdoses they witnessed likely occurred in public settings, where street-based discrimination and violence may further intensify their mental health burden.

Studies conducted in other settings, where people might be more likely to inject or use drugs in private spaces, may yield different results. For example, an analysis of death records in New Mexico found that 68.7% of overdose deaths occurred at home [36]. However, they also found that heroin-related overdose deaths were more likely than those involving prescribed opioids to occur in hotels, motels, or outdoor settings. Additionally, they found that these heroin-related overdose deaths were more likely to occur among younger individuals, males, and those showing physical signs of injection drug use, patterns that closely resemble our findings [36]. Our results should still be interpreted with caution and considered within the unique context and characteristics of the San Francisco Bay Area. Despite these limitations, our findings remain highly relevant for local public health agencies and policymakers aiming to address the overdose crisis and support one of the most disenfranchised populations, who are often on the front lines of saving lives on the streets.

## Conclusions

Nearly four in five PWID in Oakland and San Francisco, California, recently witnessed an overdose. PWID who witness overdoses are at greater risk of non-fatal overdose and may be the first to respond when those they inject with overdose. Targeting naloxone distributions to PWID who are characteristically more likely to witness overdoses may prevent additional drug-related deaths.

## Data Availability

The data that support the findings of this study are available on request from the corresponding author. The data are not publicly available due to privacy or ethical restrictions.

## Abbreviations

PWID: People who inject drugs
RDS: Respondent-driven sampling
TLS: Time-location sampling
AIC: Akaike information criterion
